# Sex Differences in Potentially Inappropriate Prescribing Among Older Adults With Multimorbidity

**DOI:** 10.1093/geroni/igab046.1296

**Published:** 2021-12-17

**Authors:** Maria Ukhanova, Sheila Markwardt, Jon Furuno, Laura Davis, Brie Noble, Ana Quiñones

**Affiliations:** 1 Oregon Health & Science University, Portland, Oregon, United States; 2 Neighborhood Health Center, Hillsboro, Oregon, United States

## Abstract

Sex differences in prescribing potentially inappropriate medications (PIMs) for various multimorbidity patterns are not well understood. This study sought to identify sex specific risk of PIMs in older adults with cardiovascular-metabolic patterns. Secondary analysis of the Health and Retirement Study interview data (2004-2014; n=6,341, ≥65 y/o) linked to Medicare claims data was conducted. Four multimorbidity patterns were identified based on the list of 20 chronic conditions and included: ‘cardiovascular-metabolic only’, ‘cardiovascular-metabolic plus other physical conditions’, ‘cardiovascular-metabolic plus mental conditions’, and ‘no cardiovascular-metabolic disease’ patterns. Presence of PIM prescribing was identified using the 2015 American Geriatrics Society Beers Criteria, limited to the list of medications to avoid in older adults. Chi-square tests and logistic regressions were used to identify sex differences in prescribing PIMs across multimorbidity patterns: (1) for PIMs overall and (2) for each PIM drug class. Results indicate that on average women were prescribed PIMs more often than men (39.4% and 32.8%, respectively). Women with cardiovascular-metabolic plus other physical patterns (Adj.OR=1.25, 95% CI: 1.07-1.45) and cardiovascular-metabolic plus mental patterns (Adj.OR=1.25, 95% CI: 1.06-1.48) had higher odds of PIM compared to men, however, there were no sex differences in PIM prescribing in the cardiovascular-metabolic only patterns (Adj.OR=1.13, 95% CI: 0.79-1.62). There was variation by sex across different PIM drug classes. Our study emphasizes the need to further reduce PIM prescribing among older adults, and identifies target populations for potential interventions to improve medication prescribing practices.

